# Aberrant ALOX5 Activation Correlates with HER2 Status and Mediates Breast Cancer Biological Activities through Multiple Mechanisms

**DOI:** 10.1155/2020/1703531

**Published:** 2020-11-10

**Authors:** Xiao Zhou, Yi Jiang, Qiuyun Li, Zhen Huang, Huawei Yang, Changyuan Wei

**Affiliations:** Department of Breast Surgery, Guangxi Medical University Cancer Hospital, Nanning, Guangxi 530021, China

## Abstract

Arachidonate lipoxygenases (ALOX) have been implicated in playing a critical role in tumorigenesis, development, and metastasis. We previously reported that ALOX12 is involved in breast cancer chemoresistance. In this study, we demonstrate that the ALOX5 activation correlates with the HER2 expression and mediates breast cancer growth and migration. We found that the ALOX5 expression and activity were upregulated in breast cancer patients, particularly in those tissues with HER2-positive. ALOX5 upregulation was also observed in HER2-positive breast cancer cells. In contrast, HER2 inhibition led to decreased expression and activity of ALOX5 but not ALOX5AP, suggesting that HER2 specifically regulates the ALOX5 expression and activity in breast cancer cells. We further demonstrated that ALOX5 is important for breast cancer biological activities with the predominant roles in growth and migration, likely through RhoA, focal adhesion, and PI3K/Akt/mTOR signaling but not epithelial mesenchymal transition (EMT). Our work is the first to report a correlation between the ALOX5 activity and HER2 overexpression in breast cancer. Our findings also highlight the therapeutic value of inhibiting ALOX5 in breast cancer, particularly those patients with the HER2 overexpression.

## 1. Introduction

Breast cancer is the most common cancer in women and the leading cause of cancer mortality worldwide [1]. Breast cancer is extensively heterogenous in pathological feature and molecular profiling, and therefore treatment regimen for each patient [2, 3]. Generally, surgical removal and chemotherapy are widely used in the clinical management of patients with breast cancer [4]. Patients with estrogen receptor- (ER-) positive are treated with adjuvant endocrine therapy such as tamoxifen, and patients with human epidermal growth factor receptor 2- (HER2-) positive are treated with adjuvant targeted therapy such as herceptin [5, 6]. However, most patients do not respond to treatment and relapse [7]. The alternative therapeutic strategy is required to improve the clinical outcome of breast cancer patient.

Human lipoxygenases (LOX) are a family of six enzymes that have been implicated in playing a key role in tumorigenesis and development via regulating the arachidonic acid (AA) pathway [8, 9]. LOX, such as ALOX12 and ALOX5, have been shown to stimulate oncogenes' expression and display mitogenic and chemotactic effects in various types of cancer cells [10–12]. In our previous work, ALOX12 was elevated in breast cancer and protected breast cancer cell from chemotherapy-induced growth arrest and apoptosis [13]. This study focused on the investigation of ALOX5's role in breast cancer. ALOX5 metabolites AA to 5-hydroperoxy-eicostetraeoic acid (5-HpETE) which further converts to 5-hydroxyicosatetraenoic acid (5-HETE) and leukotriene A4(LTA4). LTA4 is an active lipid critically involved in inflammation and cancer [14]. Studies reveal that ALOX5 and its metabolites 5-HETE are overexpressed in cancers and confer growth advantage [15, 16]. Rahul et al.'s work suggests that serum ALOX5 can serve as a progressive protein marker for breast cancer [17]. Similarly, the aberrant expression of the 5-lipoxygenase-activating protein (5-LOXAP) which activates ALOX5 has prognostic and survival significance in patients with breast cancer [18]. This study systematically investigated the expression and functions of ALOX5 in breast cancer tissues and cells and the relationship between ALOX5 and HER2, addressing the therapeutic potential of inhibiting ALOX5 in breast cancer.

## 2. Materials and Methods

### 2.1. Primary Tissues, Cell Lines, Drug, and Antibodies

Normal (*n* = 10) and malignant (*n* = 30) samples were obtained from the Tissue Repository Department of the Affiliated Cancer Hospital of Guangxi Medical University. The patients' characteristics are summarized in Supplementary Table 1. Human breast cancer cell lines were obtained from The Cell Bank of Type Culture Collection of Chinese Academy of Sciences. Cells were maintained under the same culture conditions as described in our previous studies [13, 19]. Zileuton (Cayman, USA) was reconstituted in dimethyl sulfoxide (DMSO) and stored at aliquot in -20°C. Antibodies against p-PI3K p85 (Y199), p-Akt (S437), p-mTOR(S2481), p-paxillin (T118), p-FAK(Y397), and their total proteins were purchased from Cell Signaling Inc. Antibody for ALOX5 was obtained from ThermoFish Scientific Inc.

### 2.2. Immunohistochemical Analysis

Immunohistochemical analysis was performed on the frozen tissue sections using the standard protocol including fixation, antigen retrieval, and staining [13]. Anti-ALOX5 and designated secondary antibodies were purchased from ThermoFish Scientific Inc. The signal was developed using the Pierce DAB (3, 3-diaminobenzidine) Substrate kit.

### 2.3. Cellular Activity Assays

Cell proliferation was carried out using the BrdU Cell Proliferation Assay kit (Cell Signaling, US). Cell apoptosis was determined by firstly staining apoptotic cells using annexin V-FITC and propidium iodide (PI) apoptosis kit (Sigma-Aldrich, USA), followed by analyzing cells using flow cytometry (Beckman Coulter, USA). Cell migration was determined using the Boyden chamber assay (Cell Biolabs Inc, USA). Briefly, cells together with zileuton were seeded in the top of the insert in serum-free media. Media with serum was placed in the well below. Nonmigratory cells were removed with a cotton swab. Migratory cells move through the pores below were stained with Giemsa (Sigma) and counted under microscope. Specific condition for each experiment is described in the figure legends.

### 2.4. ELISA Assays

The intracellular level of ALOX5 and 5-HETE was determined using total cell or tissue lysates and was measured using kits (ALOX5 ELISA kit and 5-HETE ELISA kit) from MyBioScource Inc. according to the manufacturer's instructions. These assays employ the quantitative sandwich enzyme immunoassay technique by using antibody specific for ALOX5 or 5-HETE.

### 2.5. Transfection

Cells were transfected with 100 nM siRNA using the Lipofectamine Transfection Reagent (Invitrogen, USA) according to the manufacturer's instructions. After transfection for 6 hours, the transfection media was replaced with culture media. The silencing of the HER2 or ALOX5 expression was assessed by Western blotting at 48 h posttransfection. The human HER2 siRNA sequence was CUA CAA CAC AGA CAC GUU U. Human ALOX5a and ALOX5b siRNA sequences were GUA CAG GAA GGG AAC AUU UUU and UUC AUG UCC UUC CCU UGU AAA. Nontargeting siRNA with at least four mismatches with all known human genes was used as negative control. siRNA Oligos set was obtained from Abcam, US. Cells were harvested for analysis 48 h after transfection.

### 2.6. Statistical Analyses

**Figure 1:**
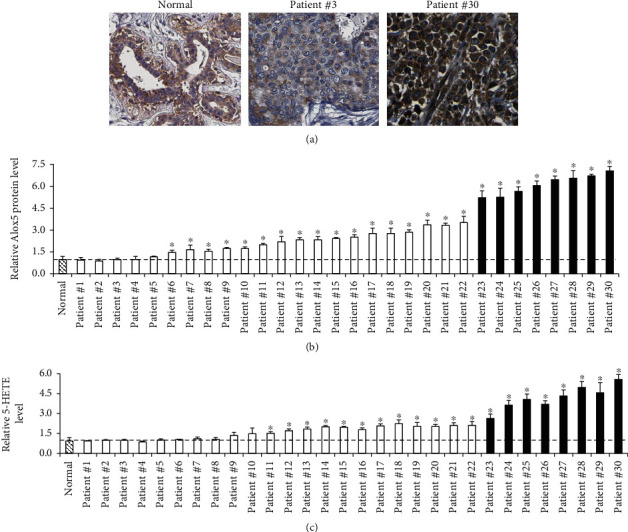
The ALOX5 expression and activity are upregulated in breast cancer. (a) Representative immunohistochemical analysis of ALOX5 on the normal breast tissue and breast cancer tissue from two patients (#3 and #30). The intracellular level of ALOX5 (b) and 5-HETE (c) in the normal breast tissue (*n* = 10, results were shown as average of ten samples) and breast cancer tissue (*n* = 30, results were shown as individual of each sample). Column filled with black was HER2-positive, and column without filling was HER2-negative. ALOX5 and 5-HETE in normal breast tissues were set as 1. ^∗^*p* < 0.05, compared to normal.

**Figure 2:**
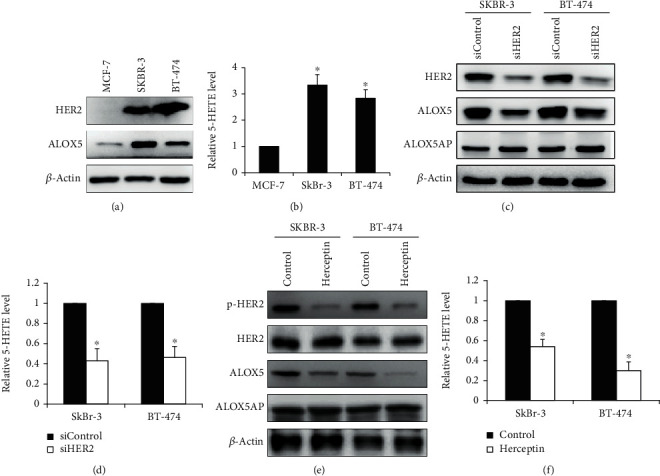
The HER2 overexpression promotes the ALOX5 expression and activity in breast cancer cells. (a) Representative Western blotting image of HER2 and ALOX5 in MCF-7, SKBR3, and BT-474 cells. (b) SKBR3 and BT-474 have higher 5-HETE level than MCF-7. (c) Representative Western blotting image of HER2, ALOX5, and ALOX5AP in SKBR3 and BT-474 cells after HER2 siRNA knockdown. Cells were harvested for analysis at 48 h posttransfection. (d) HER2 knockdown significantly decreases the 5-HETE level in breast cancer cells. (e) Representative Western blotting image of p-HER2, HER2, ALOX5, and ALOX5AP in SKBR3 and BT-474 cells exposed to 10 *μ*M zileuton. (f) Herceptin (20 *μ*g/ml) significantly decreases the 5-HETE level in breast cancer cells. ^∗^*p* < 0.05, compared to MCF-7, siControl, or control.

**Figure 3:**
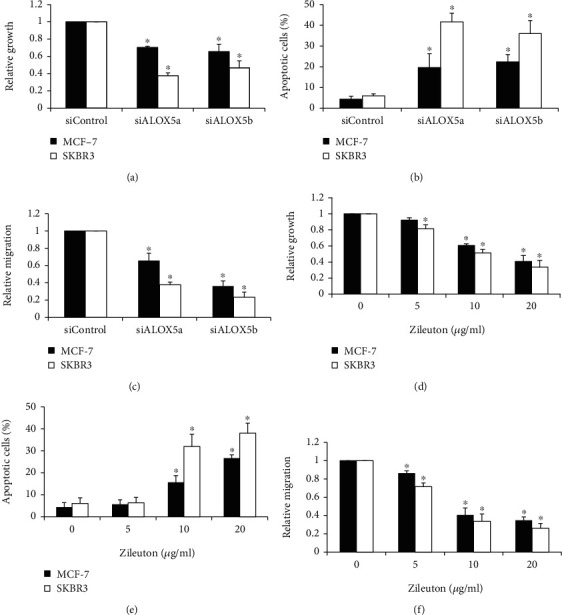
ALOX5 inhibition is active against breast cancer cells. ALOX5 inhibition via siRNA or zileuton significantly inhibits proliferation (a and d), induces apoptosis (b and e), and suppresses migration (c and f) in MCF-7 and SKBR3 cells. Proliferation and apoptosis were determined after 3 days drug treatment. Migration was determined after 8 hours of drug treatment. Transfected cells were harvested at 48 h posttransfection for proliferation, apoptosis, and migration assays. ^∗^*p* < 0.05, compared to siControl or control.

**Figure 4:**
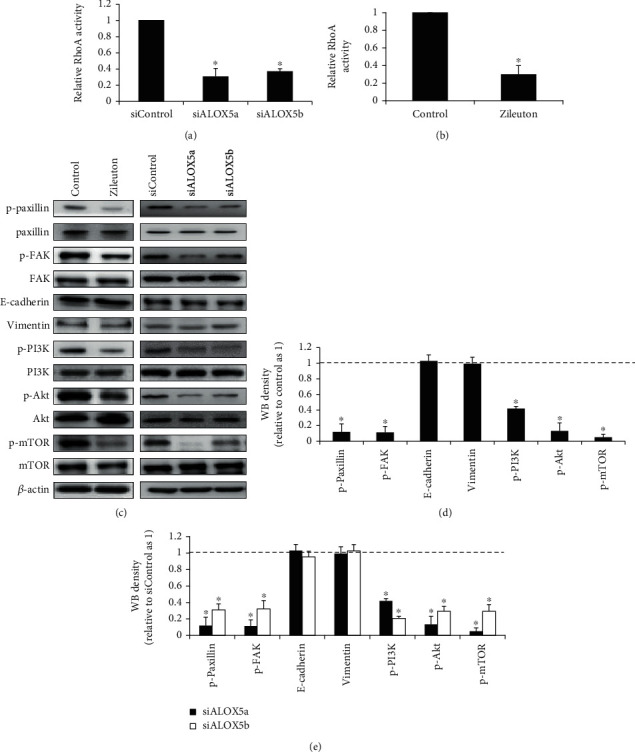
ALOX5 inhibition suppresses RhoA, focal adhesion, and PI3K/Akt/mTOR in breast cancer cells. ALOX5 inhibition via siRNA (a) and zileuton (b) significantly decreases the RhoA activity in SKBR3 cells. Representative image (c) and quantification (d) of Western blotting of p-paxillin, p-FAK, E-cadherin, vimentin, p-PI3K, p-Akt, and p-mTOR in SKBR3 cells exposed to zileuton or transfected with siALOX5. Western blot analysis was performed using SKBR3 cells after 1-day zileuton treatment or the transfected cells at 48 h posttransfection. ^∗^*p* < 0.05, compared to control or siControl.

All data are expressed as mean and standard deviation. For Figure 1, a one-way analysis of variance (ANOVA) and the post-hoc Tukey honestly significant difference (HSD) test was used. For Figures 2–4, Student's *t* test was used. These figures were obtained from three independent experiments.

## 3. Results

### 3.1. The ALOX5 Expression and Activity Are Upregulated in Breast Cancer

We first analyzed the overall protein levels of the ALOX5 in the cohort of ten normal breast tissue and breast cancer tissues from thirty patients using both immunohistochemical and ELISA approaches. Compared to normal breast tissues, we observed the increased ALOX5 staining in the breast cancer tissue from some but not all patients (Figure 1(a)). We then averaged the ALOX5 level of 10 normal breast tissues and compared it with each individual breast cancer tissue. Consistently, ELISA analysis demonstrated the significant increase on the intracellular protein level of ALOX5 among 25 out of 30 breast cancer tissues (Figure 1(b), indicated by an asterisk ∗), indicating that the ALOX5 level is upregulated in 80% of all breast cancer patients screened. In addition, the increasing fold change varied among breast cancer tissues, ranging from 1.5 to 7 times. We next classified the breast cancer tissues with more than 5-fold increase on ALOX5 as the breast cancer tissue ALOX5^high^.

Notably, the clinicopathological information shown in Supplementary Table 1 demonstrated that the breast cancer tissue ALOX5^high^ was all HER2-positive. We did not observe the correlation between the ALOX5 expression and other clinicopathological features, such as ER and PR. The selective synthesis of 5-HETE is dependent on ALOX5 and therefore serves as the function of the ALOX5 activity [20]. We found the significant increase on 5-HETE production among 20 out of 30 breast cancer tissues compared to the normal breast tissue (Figure 1(c)), demonstrating the increased ALOX5 activity. Particularly, the breast cancer tissues with significant increased 5-HETE production demonstrated minimal 2-fold increase on the ALOX5 level. Taken together, these results suggest that the ALOX5 expression and activity are upregulated in breast cancer and are associated with the HER2 expression.

### 3.2. ALOX5 Is Regulated by HER2 in Breast Cancer

We next investigated the expression of ALOX5 and HER2 in breast cancer cells. We employed the SKBR3 and BT-474 cell line which are HER2-overexpressing breast cancer cell line and MCF-7 cell line which is negative for HER2 [21]. Western blotting analysis confirmed the absence of HER2 in MCF-7 and presence of HER2 in SKBR3 and BT-474 (Figure 2(a)). ALOX5 and 5-HETE levels were also higher in SKBR3 and BT-474 cells compared to MCF-7 cells (Figures 2(a) and 2(b)). To determine the correlation of ALOX5 and HER2, we analyzed the effects of HER2 inhibition on the expression and activation of ALOX5 using both genetic and pharmacological inhibition methods. HER2 siRNA knockdown decreased the ALOX5 level and 5-HETE production in SKBR3 and BT-474 cells (Figures 2(c) and 2(d)). In contrast, HER2 siRNA knockdown did not affect the ALOX5AP level. Herceptin is a recombinant humanized monoclonal antibody against HER2 by inducing impairment of HER2 heterodimerization and consequent blockade of downstream signaling events [22]. Consistent with the previous report [23], we observed the decreased HER2 phosphorylation without change in the total HER2 expression in cells treated with herceptin (Figure 2(e)). We found that herceptin treatment decreased the ALOX5 expression and 5-HETE production but not the ALOX5AP expression (Figures 2(e) and 2(f)). These results demonstrated that HER2 specifically regulates the ALOX5 expression and activity in breast cancer cells.

### 3.3. ALOX5 Depletion Is Active against Breast Cancer Cells

To investigate the role of ALOX5 in breast cancer cells, we determined the effects of ALOX5 inhibition on the growth, survival, and migration of cells. ALOX5 knockdown using two independent siRNAs consistently inhibited proliferation up to ~60% and migration up to ~80% and induced apoptosis up to 40% in MCF-7 and SKBR3 cells (Figures 3(a)–3(c)). We further found that SKBR3 is more sensitive to ALOX5 inhibition than MCF-7. It is worth noting that the decreased growth, survival, and migration in breast cancer cells are not limited to siRNA knockdown; the treatment of a selective ALOX5 inhibitor zileuton resulted in a similar phenotype in breast cancer cells in a dose-dependent manner (Figures 3(d)–3(f)). These results demonstrate that ALOX5 is important for the breast cancer cell biological function with predominant roles in growth and survival.

### 3.4. ALOX5 Inhibition Suppresses RhoA, Focal Adhesion, and PI3K/Akt/mTOR Signaling in Breast Cancer Cells

Since RhoA GTPase and focal adhesion play a central role for all types of cell migration [24], we asked whether ALOX5 inhibition affected the RhoA signaling and focal adhesion. We examined the RhoA activity and the phosphorylation of two essential focal adhesion molecules: paxillin and focal adhesion kinase (FAK) [25], in the cells after ALOX inhibition. We found that ALOX5 inhibition by zileuton and siRNA significantly decreased the RhoA activity in SKBR3 cells (Figures 4(a) and 4(b)). ALOX5 inhibition also suppressed the phosphorylation of paxillin and FAK (Figures 4(c)–4(e)). We did not observe any changes on the expression level of E-cadherin and vimentin which are critically involved in epithelial mesenchymal transition (EMT). Furthermore, we found that ALOX5 inhibition by siRNA and zileuton significantly decreased the phosphorylation of PI3K, Akt, and mTOR. Taken together, we demonstrate that ALOX5 inhibition suppresses RhoA, focal adhesion, and PI3K/Akt/mTOR signaling in breast cancer cells without affecting EMT.

## 4. Discussion

Increasing evidence have demonstrated that human cancer tissues display aberrant overexpression and activation of ALOX enzymes, particularly ALOX5 and ALOX12, to promote tumorigenesis, angiogenesis, and tumor cell invasiveness possibly via modulating tumor cell oxidative and arachidonic acid metabolism, oncogenic signaling pathways, and inflammatory [10, 11, 26, 27]. It has been suggested that ALOX inhibition represents a promising target for cancer chemoprevention [16, 28]. However, the role of ALOX in breast cancer remains much unknown. This study firstly demonstrated that the ALOX5 expression elevated breast cancer tissues compared to control (Figures 1(a) and 1(b)), which is supported by the report that the mean concentration of serum ALOX5 is significantly higher in breast cancer patients than similar age as the control group [17]. In addition, the serum level of ALOX5 among individual breast cancer patients displays the same trend as what we observed in breast cancer tissues; only some but not all breast cancer samples screened with the aberrant ALOX5 expression. The expression pattern of ALOX5 in breast cancer is different from ALOX12 as shown in our previous study that ALOX12 is persistently upregulated in all tested breast cancer samples regardless of subtypes, cellular origin, and genetic background [13]. Consistently, ALOX5AP which is necessary for the ALOX5 activation via facilitating the docking of AA to ALOX5 has been found to be overexpressed in breast cancer, particularly in aggressive tumors [18]. In addition, both ALOX5 and ALOX5AP have prognostic and survival significance in breast cancer patients [17, 18], suggesting that the elevated level of the expression is functional. This is supported by our observation that the 5-HETE level was significantly increased in breast cancer tissues (Figure 1(c)).

Rahul et al. found that HER2, PR ,and ER status and other clinical histopathological features did not affect the serum level of ALOX5 [17]. Our findings identified the variation in the tissue ALOX5 level with HER2 status but not RP or ER status (Figure 1 and Supplementary Table 1) and suggested that the expression is likely to be associated with the HER2 overexpression. Further studies confirmed that the ALOX5 expression and activity are regulated by the HER2 overexpression as inhibition of the HER2 activation or expression in breast cancer cells resulted in decreased activation and expression of ALOX5 but not ALOX5AP (Figure 2). Cytosolic phospholipase A2 (cPLA2) is another critical regulator of AA metabolism and has been reported be associated with the HER2 overexpression in breast cancer [29]. The overexpression of HER2 is found in approximately 30% of breast cancer patients and correlates with poor clinical outcome [30]. Our findings together with previous report highlight the importance of the AA pathway in HER2-positive breast cancer.

Functional analysis of ALOX5 via genetic and pharmacological approaches on HER2-negative and -positive cells demonstrated that ALOX5 was involved in growth, survival, and migration in breast cancer cells regardless of HER2 status, although HER2-positive cells are more sensitive to ALOX5 inhibition than HER2-negative cells (Figure 3). This is consistent with the previous work that the ALOX5 inhibitor induces apoptosis and growth arrest in human breast cancer cell lines [17]. In addition, we extend the previous studies by showing that migration was the most predominant role of ALOX5 in breast cancer as ALOX5 inhibition led to the largest degree of migration inhibition compared to growth arrest and apoptosis induction (Figure 3). This is well correlated with the finding that HER2-positive breast cancer tends to be more invasive [31]. We further demonstrated that ALOX5 inhibition suppressed breast cancer migration via decreasing the RhoA activity and subsequently inhibiting focal adhesion formation (Figure 4). This is the first evidence to show the association between ALOX5 and RhoA/focal adhesion signaling in tumor cells. In addition, ALOX5 inhibition also suppressed the PI3K/Akt/mTOR signaling in breast cancer cells (Figure 4). HER2 amplification in tumors activates the PI3K/Akt signaling [32]. We show that the PI3K/Akt activation can be suppressed by targeting ALOX5 in breast cancer cells.

In conclusion, our work demonstrated that the aberrant ALOX5 activation is associated with the HER2 overexpression and mediates breast cancer growth, migration, and survival. The selective ALOX5 inhibitor zileuton is an FDA-approved drug for the prevention and chronic treatment of asthma [33]. The efficacy of zileuton alone and its combination with anticancer drugs for the treatment of lung cancer are under investigation in clinical settings (ClinicalTrials.gov Identifier: NCT00056004 and NCT00070486). Our preclinical findings provide rational for evaluating zileuton in breast cancer patients, particularly in HER2-positive patients.

## Data Availability

Data will be made available on request.

## Supplementary Materials

Supplementary Materials Supplementary Table 1: breast cancer patients' characteristics.
