# Supplementary material for: Aberrant ALOX5 Activation Correlates with HER2 Status and Mediates Breast Cancer Biological Activities through Multiple Mechanisms
Source: Biomed Res Int. 2020 Nov 10;2020:1703531. doi: 10.1155/2020/1703531 (PMC7673939; doi:10.1155/2020/1703531)
Supplement: Supplementary Materials — Supplementary Table 1: breast cancer patients' characteristics. [file 1703531.f1.zip › Supplementary description.doc]

**Aberrant ALOX5 activation correlates with HER2 status and mediates breast cancer biological activities through multiple mechanisms**

**Supplementary Table 1: Breast cancer patients’ characteristics**

Notes: Tumor size, T1 is smaller than 20mm; T2 is larger than 20 mm but less than 50 mm; T3 is larger than 50 mm. Lymph node, N1- The cancer has spread to 1 to 3 axillary lymph nodes; N2- The cancer has spread to 4 to 9 axillary lymph nodes; N3- The cancer has spread to 10 or more axillary lymph nodes. HER2, human epidermal growth factor receptor 2; ER, estrogen receptor; RP, progesterone receptor.
