# Supplementary material for: Aberrant ALOX5 Activation Correlates with HER2 Status and Mediates Breast Cancer Biological Activities through Multiple Mechanisms
Source: Biomed Res Int. 2020 Nov 10;2020:1703531. doi: 10.1155/2020/1703531 (PMC7673939; doi:10.1155/2020/1703531)
Supplement: Supplementary Materials — Supplementary Table 1: breast cancer patients' characteristics. [file 1703531.f1.zip › Supplementary file.doc]

**Aberrant ALOX5 activation correlates with HER2 status and mediates breast cancer biological activities through multiple mechanisms**

**Supplementary Table 1: Breast cancer patients’ characteristics**

|  | Age | Clinical stage | Tumor size | Node | HER2 | ER | PR |
| --- | --- | --- | --- | --- | --- | --- | --- |
| Patient#1 | 67 | IV | T2 | N1 | **-** | **-** | **-** |
| Patient#2 | 69 | IV | T1 | N1 | **-** | **-** | **+** |
| Patient#3 | 45 | IV | T2 | N2 | **-** | **+** | **-** |
| Patient#4 | 76 | IV | T2 | N2 | **-** | **+** | **+** |
| Patient#5 | 57 | IV | T1 | N1 | **-** | **+** | **-** |
| Patient#6 | 54 | IV | T2 | N2 | **-** | **-** | **++** |
| Patient#7 | 51 | IV | T1 | N1 | **-** | **-** | **-** |
| Patient#8 | 47 | IV | T2 | N3 | **-** | **-** | **+** |
| Patient#9 | 48 | IV | T3 | N2 | **-** | **+** | **-** |
| Patient#10 | 78 | IV | T3 | N2 | **-** | **+** | **-** |
| Patient#11 | 81 | IV | T1 | N1 | **-** | **-** | **+** |
| Patient#12 | 56 | IV | T3 | N3 | **-** | **+** | **+** |
| Patient#13 | 58 | IV | T1 | N1 | **-** | **-** | **-** |
| Patient#14 | 54 | IV | T1 | N1 | **-** | **+** | **+** |
| Patient#15 | 67 | IV | T2 | N2 | **-** | **-** | **+** |
| Patient#16 | 34 | IV | T3 | N2 | **-** | **+** | **+** |
| Patient#17 | 32 | IV | T2 | N2 | **-** | **-** | **-** |
| Patient#18 | 38 | IV | T3 | N2 | **-** | **-** | **-** |
| Patient#19 | 43 | IV | T2 | N2 | **-** | **+** | **-** |
| Patient#20 | 49 | IV | T2 | N3 | **-** | **+** | **+** |
| Patient#21 | 56 | IV | T3 | N3 | **-** | **+** | **+** |
| Patient#22 | 57 | IV | T1 | N1 | **-** | **+** | **+** |
| Patient#23 | 65 | IV | T3 | N2 | **+** | **+** | **-** |
| Patient#24 | 67 | IV | T2 | N3 | **+** | **+** | **-** |
| Patient#25 | 63 | IV | T2 | N3 | **+** | **-** | **-** |
| Patient#26 | 64 | IV | T2 | N3 | **+** | **-** | **-** |
| Patient#27 | 54 | IV | T2 | N3 | **+** | **+** | **-** |
| Patient#28 | 49 | IV | T2 | N3 | **+** | **-** | **-** |
| Patient#29 | 56 | IV | T3 | N2 | **+** | **+** | **-** |
| Patient#30 | 43 | IV | T1 | N1 | **+** | **-** | **+** |

Notes: Tumor size, T1 is smaller than 20mm; T2 is larger than 20 mm but less than 50 mm; T3 is larger than 50 mm. Lymph node, N1- The cancer has spread to 1 to 3 axillary lymph nodes; N2- The cancer has spread to 4 to 9 axillary lymph nodes; N3- The cancer has spread to 10 or more axillary lymph nodes. HER2, human epidermal growth factor receptor 2; ER, estrogen receptor; RP, progesterone receptor.
